# An age friendly cross-sector program to address health-related social needs and prevent avoidable hospitalization

**DOI:** 10.1093/geroni/igaf122.3155

**Published:** 2025-12-31

**Authors:** Jose Martinez Escudero, Arielle Basch

**Affiliations:** JASA, New York, New York, United States; JASA, Litchfield, Connecticut, United States

## Abstract

Older adults face post-hospitalization risks including medication complexity, barriers to follow-up care, and limited understanding of how to manage health conditions at home. These challenges are exacerbated for patients with limited English proficiency, lower health literacy, and complex social needs. JASA Aging Services has partnered with hospitals and health plans in NYC to create an age-friendly hospital and home-based transitional care model to address social needs and reduce readmissions. It includes several innovative elements: age-friendly care across settings driven by “what matters” to the patient; home-based education to build trust and learn about the patient’s environment; an in-language, culturally appropriate model led by international medical graduates with extensive clinical knowledge and concordance of (10+) languages and cultures with patients. The program provides education, medication reconciliation, and vital sign monitoring while addressing social needs. The model shows early signs of success. Between February 1, 2024 and January 31, 2025, 1353 patients received JASA’s transitional care services of which 83% were connected to essential social services, 56% completed a healthcare proxy form, and 72% received essential support with their medications. We achieved a 38% reduction in readmissions and patients were 21% more likely to engage with a PCP compared to baseline data. Patient satisfaction scores exceed 97%.

